# The Urgent Need for International Action for Anti-aging and Disease Prevention

**DOI:** 10.14336/AD.2019.1230

**Published:** 2020-02-01

**Authors:** Robert Chunhua Zhao, Ilia Stambler

**Affiliations:** ^1^Center of Excellence in Tissue Engineering, Department of cell biology, Institute of Basic Medical Sciences Chinese Academy of Medical Sciences, School of Basic Medicine Peking Union Medical College, Beijing, China; ^2^The Geriatric Medical Center "Shmuel Harofe", Beer Yaakov, affiliated to Sackler School of Medicine, Tel-Aviv University, Tel-Aviv, Israel; ^3^International Society on Aging and Disease (ISOAD), Fort Worth, Texas, USA.

## The establishment of the Executive Committee on Anti-Aging and Disease Prevention

There is a growing consensus that researching and developing therapeutic interventions into degenerative aging processes is a necessary condition for improving the health and longevity of the rapidly aging global population Thus, the research and development of therapies against degenerative aging processes (anti-aging) and for prevention of major aging-related diseases is a necessary condition for alleviating the severe economic, healthcare and humanitarian challenges of the global aging society And therefore, promoting the research and development in the field of anti-aging and aging-related disease prevention is becoming an urgent national and international task [1,2] How can the field of anti-aging and disease prevention be promoted globally to solve the challenge of bringing effective, safe and accessible anti-aging and preventive therapies to the world as soon as possible? A significant further step was taken toward the solution of this challenge with the establishment of the new Executive Committee on Anti-aging and Disease Prevention, a joint effort of UNESCO and China World Peace Foundation.

The Executive Committee on Anti-aging and Disease Prevention was established in the framework of Science and Technology, Pharmacology and Medicine Themes under an Interactive Atlas along the Silk Roads, UNESCO. The committee inauguration took place during the 2nd (Beijing) Annual International Biomedical Health Conference and the 1st Academician Forum of Transnational Biomedical Field, in Beijing, on December 16, 2019 (http://www.isoad.org).

The fact that the new Executive Committee on Anti-aging and Disease Prevention was initiated in China is not a coinaccidence. The problem of aging is common for the entire world. Yet, for China, the problem of rapid population aging is especially pressing, as its population aging rate and aging dependency growth are among the fastest in the world, with the absolute number of the elderly being the largest in the world (about 167 million or 12% of the country population over 65 in 2019, which will grow to as much as 330 million or about 26% by 2050) [3]. At the same time, China has at its disposal powerful technological, scientific and social capabilities to address the challenge.

The vital need to address the aging challenge has been increasingly recognized by the Chinese government and stakeholders in China. The year 2019 and the next year will be crucial years for China to formulate the14^th^ Five-year Plan, and to achieve the first centenary goal and move towards the second centenary goal, in the face of the unprecedented social, technological and demographic changes that have never been experienced in a century. At such a time, the 2^nd^ Annual International Biomedical Health Conference convened in Beijing, responding to President Xi’s call for building a healthy China and a community with shared future for mankind with practical actions!

The conference responded to the increasingly serious crisis of chronic diseases and aging worldwide. Therefore, the conference focused on the global problems of aging and the related rise of chronic major diseases and discussed how biotechnology can creatively provide solutions for human health, in particular in old age. As the aging-related health problems increase, so do increase the capabilities of biomedical science and technology to solve those problems. For example, 5G technology will allow us to develop from information sharing to internet of things (IOT). Artificial intelligence (AI) will greatly improve our ability to understand and solve complex problems, while the breakthroughs in stem cell technologies and other geroprotective therapies will allow us to redefine healthy aging and to completely treat major diseases.

In order to implement the great idea of a community with a shared future of mankind, the United Nations Educational, Scientific and Cultural Organization supported the establishment of a professional association on anti-aging research in China through Science and Technology, Pharmacology and Medicine Themes under an Interactive Atlas along the Silk Roads, which was named the Executive Committee on Anti-Aging and Disease Prevention, UNESCO.

In order to support the work of the committee, Institute of Biophysics at Chinese Academy of Sciences, Institute of Basic Medicine at Chinese Academy of Medical Sciences, and Shanghai University jointly established the International Joint Academy of Biomedicine as the scientific research base of the International Society on Aging and Disease (ISOAD).

The committee and affiliated organizations will invite prominent scientists in the field of biomedicine from all over the world to join the committee on behalf of the scientific research institutes in China and internationally, and carry out this project under an Interactive Atlas along the Silk Roads, UNESCO, so as to promote the latest research achievements and technologies of member states to the world. The committee will form and promote policy recommendations, educational programs and scientific and cultural projects to advance the field of anti-aging and disease prevention worldwide, at the national and international levels.

Science, technology and health are important cornerstones for world peace and human prosperity. By promoting the healthy longevity for the global population, the committee will contribute to the task of global cooperation, peace and human wellbeing.

Only by relying on the full win-win cooperation of global scientists, and relying on the standardized technical services of various countries, can the great dream of a healthy China and the great goal of a healthy world be achieved, under the active support by the policies in biotechnology and biomedical industry!

The conference and its goals were strongly supported by China’s National Development and Reform Commission and other units, with full cooperation of personnel from the biotechnology and biomedical industry all over the world, and with full-hearted active participation by academicians, specialists and peers from around the world.

It is hoped that through holding this conference and the further actions of the committee, the international cooperation will be enhanced, and more advanced scientific and technological achievements will be shared and implemented.

It is strongly believed that through the efforts of all of us, all the conference participants, all the collaborators, as well as the general public, can benefit from the results of anti-aging research, which has been the real intention for holding this conference and for the establishment of the Executive Committee on Anti-aging and Disease Prevention.

Special thanks for implementing this initiative go to Prof. Ante Glibota, Vice-president of the European Academy of Sciences, Arts and Humanities (EASAH), and Prof. Robert Chunhua Zhao, the present Chairman of the International Society on Aging and Disease (ISOAD) who will chair the committee, as well as the excellent and self-less scientists from around the world who helped with the establishment of the Executive Committee on Anti-Aging and Disease Prevention, UNESCO, in China.

The conference participants formed the core of the Executive Committee on Anti-aging and Disease Prevention that will be further expanded and reinforced in time. The present committee members include 18 experts from 16 countries and regions: Holly M. Brown-Borg (University of North Dakota, USA), Antonio Cano (University of Valencia, Spain), Calogero Caruso (University of Palermo, Italy), Sasanka Chakrabarti (Maharishi Markandeshwar University, India), Georgina Ellison-Hughes (King's College London, UK), Eric Gilson (Nice University, France), Ante Glibota (European Academy of Sciences, Arts and Humanities, France), Armand Keating (University of Toronto, Canada), Lee Wei Lim (University of Hong Kong, China), Mitsuo Maruyama (National Center for Geriatrics and Gerontology, Japan), Kyung-Jin Min (Inha University, South Korea), Alexey Moskalev (Syktyvkar State University, Russia), Amanda Salis (The University of Sydney, Australia), Ilia Stambler (Shmuel Harofe Geriatric Medical Center, Israel), Huanxing Su (University of Macau, China), Brun Ulfhake (Karolinska University Hospital, Sweden), Robert Chunhua Zhao (Institute of Basic Medical Sciences Chinese Academy of Medical Sciences & School of Basic Medicine Peking Union Medical College, China), and Kunlin Jin (University of North Texas Health Science Center, USA).

In addition to the international speakers, a distinguished cohort of prominent Chinese scientists, officials and leaders of industry presented at the conference, including the vice director Hao Chang of International Cooperation Center of National Development and Reform Commission, the chairman Yu Liang of China Health Industry Investment Fund Management, the president Xizhao Dou of National Association of Health Industry, the academician Chengyu Xiong from the Academy of Europe, Tsinghua University, Xiangdong Tan, chairman of Shanghai Magnolia Tan Jiazhen Life Science Development Foundation, the senior Vice-Mayor Linbang Lv of Lanzhou Municipal People’s Government, the district governor Hui Deng of Fucheng District, Mianyang City (China Science and Technology City), Depei Liu, Academician of Chinese Academy of Engineering, Xiangmei Chen, the Honorary Director of Department of Kidney Disease of the PLA General Hospital, Runsheng Chen, Academician of Chinese Academy of Sciences, Changsheng Liu, Academician of Chinese Academy of Sciences, and other distinguished participants.

Based on the world-class expertise of the participants, the UNESCO Executive Committee on Anti-Aging and Disease Prevention was launched, and the International Joint Academy of Biomedicine was established.

## The urgent need to develop policies, guidelines and action plans to advance anti-aging and disease prevention globally

The launch of the UNESCO Executive Committee is a further step along the road toward the global advancement of anti-aging and preventive biomedical research, development and application. The road will be long as many capacities still need to be built and many tasks implemented for the field to live up to its potential. Thus, there is an urgent need to develop action plans and roadmaps, policies and guidelines, consensus positions and educational materials, evaluation criteria and regulatory frameworks, to advance anti-aging and disease prevention worldwide. Such items are still in short supply both at the national and international levels. We hope that through international cooperation, in particular via establishing international expert task forces, these items may be created and disseminated.

The reasons to advance the medical research and development (R&D) on anti-aging and disease prevention should be made clear to all, at all levels, from the general public to the decision makers. All should be educated about this R&D, and its vital potential to provide real solutions to prevent major chronic diseases and to improve the health, longevity and quality of life for the elderly population globally.

This R&D is already supported by scientific proofs of concept, involving the evidential increase in healthy lifespan in animal models and the emerging technological capabilities to intervene into fundamental aging processes. There are also experimental proofs of the possibility to prevent multiple aging-related diseases by intervening into the aging process as the main contributing factor common to all these diseases, including positive experimental results in humans [4]. Any reinforcement of such R&D will lead to additional cumulative reinforcements, speed up the translation of basic research to clinical practice and facilitate the faster and wider distribution of the R&D results for the public. Such R&D solutions are preventative in their nature and are likely to decrease the susceptibility of the elderly to both non-communicable and communicable diseases. Therefore, such means will enable a significant increase in the expectation of healthy, productive and creative life for the aged population.

The innovative, applied results of such research and development will lead to sustainable solutions for a large array of age-related medical and social challenges, that may be globally applicable. The most important of them are the savings in healthcare for chronic age-related diseases and increase in the period of active and productive employment for the elderly population. This will result in their diminished dependence on external entities and the corresponding release of resources for further social and economic development and growth. As it is known, the vast majority of health expenditures and loss of productivity occur in the late development stages of chronic aging-related diseases. Therefore, any postponement of this period by early detection and prevention will create vast humanitarian and economic benefits, both for the individual and the society as a whole [5]. Excellence in this field will ensure competitive advantage for the countries involved, as well as the basis for broad international collaboration, insofar as the aging challenge is globally relevant.

Due to these reasons, the investments into the biomedical research and development on anti-aging and prevention of aging-related diseases, can be the most beneficial and profitable for both national and global economy compared to any kind of fundamental or applied research generally and biomedical research in particular. Conversely, the lack of investments into the field can contribute to a future crisis that may be due to the lack of preparedness of the healthcare and welfare systems to provide worthy and sufficient services for the elderly, the lack of adequate solutions for the prevention of systemic economic collapse, as well as for the equitable social and economic inclusion of the elderly.

These reasons for the advancement of the anti-aging and preventive biomedicine still need to become more strongly articulated and disseminated, to bring them to the attention and action of the entire society. International expert communities and organizations can have a pivotal role for such articulation and dissemination.

The conference furthered this articulation, addressing particular issues involved. The conference participants presented on the various aspects of anti-aging and disease prevention, and related contexts, generally showing the feasibility of improving health in old age by the use of advanced biotechnology. The main topics included: Development of stem cell-based therapies and treatment of aging-related diseases; frontier science and technology of preventive medicine and personalized therapy; integrated development and policy research of new medical technology and health industry; scientific and technological progress in biological anti-aging field and health education in aging society.

Within this range, the UNESCO committee members addressed specific areas and topics. Altogether they presented a wide front of past achievements, current work in progress and directions for future advancement for the field of anti-aging and disease prevention.

Holly Brown-Borg (USA) provided a thorough overview of the current approaches to targeting the biological mechanisms of aging, constituting the fundamentals of the geroscience strategy for the preventive healthcare for the aged, i.e. measuring and therapeutically targeting the main hallmark mechanisms of aging to prevent multiple age-related diseases (multi-morbidity) and thus extend the healthy lifespan (or healthspan) of the elderly population, with the resulting healthcare, economic and humanitarian benefits.

Calogero Caruso (Italy) shared the main results of the study of longevity in Sicily, in particular the study of local centenarians, searching for the determinants of their exceptional healthy longevity and resistance to age-related diseases and co-morbidities, including biological (molecular and genetic/epigenetic) factors as well as social and life-style determinants, with the aim to develop and adopt successful healthy longevity intervention strategies for the larger population.

Antonio Cano (Spain), though communicating from outside the conference, introduced the perspective of Aging in Spain, focusing on the challenges of facing senescence with a female perspective, in particular with reference to endocrinological problems and treatments of aging-related dysfunction.

Sasanka Chakrabarti (India) gave a perspective of aging research, its challenges and prospects for the long term, including issues of transition from animal models to clinical practice, development of aging epidemiology, biomarkers and risk factors, and properly designed anti-aging clinical trials, as well as some policy recommendations, such as improving funding and professional education for the field.

Georgina M. Ellison-Hughes (UK) represented a new collaboration model in aging research, the multidisciplinary Aging Research at Kings College London (ARK) consortium. She then presented the highly promising results of their study for rejuvenating the regenerative capacity of the aged heart, one of the main causes of old age morbidity and mortality.

Eric Gilson (France), though communicating from outside the conference, introduced another collaborative model, the Inserm Transversal program on aging AGEMED (From Aged Cells to Medical Applications) including 20 French teams covering all topics of aging biology and medical transfer. This is a collaboration model that could be emulated also for international frameworks, emphasizing the need for holistic approaches and international coalition in aging research, development and clinical application.

Ante Glibota (France) reported in his speech that the European Academy of Sciences, Arts and Humanities, as UNESCO's consultants, worked closely with the relevant stakeholders for the establishment of the new UNESCO Executive Committee on Anti-Aging and Disease Prevention. He expressed the belief that enhanced international cooperation will enable the collaborators to perform thorough systemic analysis, develop actionable solutions and improve coordination and management of the medical and scientific communities to advance anti-aging technologies and biological strategies. Such international collaborative structures will help involved organizations to develop stem cell and other anti-aging therapies that can prevent diseases, alleviate human suffering and promote human health.

Armand Keating (Canada) discussed perspectives on anti-aging research, from its history to its future, with a special focus on hematological aspects of aging. In particular, he emphasized hematopoiesis as a key area of aging research, addressing key diagnostic and therapeutic issues in this research and the need for innovation and development of this area.

Lee Wei Lim (Hong Kong, China) introduced the field of electromagnetic deep brain stimulation, which may be a novel and effective therapeutic strategy for a variety of problems of aging, with potential benefits ranging from anti-depression to memory enhancement, and even potentially affecting fundamental whole-body regulatory mechanisms of aging such as hypothalamus regulation.

Mitsuo Maruyama (Japan) reported on the severe challenges of the aging society in Japan, and the national programs and centers established to address those problems, including the enhancement of biomedical research and development.

Kyung-Jin Min (South Korea) presented an overview of aging research in Korea and their institution in particular, showcasing a broad and intensive search for anti-aging drugs and other interventions in animal models, with the aim of possible translation to humans.

Alexey Moskalev (Russia) surveyed the advances in the genetics of longevity and aging, providing an exposition of biomarkers of aging and possible geroprotective interventions (including pharmacological, gene and cell therapies) under development. He also introduced several suggestions for the improvement of geriatric healthcare, such as developing new approaches for training medical personnel, creating knowledge bases for early diagnosis, prevention and treatment of age-related diseases and conditions, establishing multi-center clinical studies for age-related disease prevention, and developing panels of biomarkers of aging and geroprotectors.

Amanda Salis (Australia) made a compelling case for anti-aging dietary interventions, specifically for reducing the diseases of aging via substantial weight loss in people with overweight or obesity in Australia and around the world.

Ilia Stambler (Israel) presented the advances of anti-aging R&D regulation and policy in Israel and internationally, the critical need to further develop and implement the policies supportive of the anti-aging field, and some possible directions for their development, such as increasing funding, education and supportive regulatory measures and evaluation standards for the filed that could be designed and promoted via international organizations.

Huanxing Su (Macau, China) gave an overview of drug-discovery for aging-related neurodegenerative diseases, focusing on finding natural products and Chinese herbs to treat microinfarcts and vascular dementia.

Brun Ulfhake (Sweden) spoke about the mechanisms of the decline in muscle power and mass as the core of organismal aging, and discussed possible actionable interventions, such as diet and exercise. He emphasized that while our current and near-future research will extend our understanding of the underlying processes and pave the way for rational interventions, we need to implement our current knowledge, but never step back on scientific rigor, to combat aging and aging-associated disabilities, and further, to adapt the society to a growing population of the elderly.

Robert Chunhua Zhao (Beijing, China), in his scientific presentation, reported the results of studies with mesenchymal stem cells and their role in clinical translational medicine. He focused on some of the central problems in translating anti-aging therapies to clinical practice, with stem cells representing a major type of anti-aging therapy, in particular defining the subpopulations of stem cells as the best seed cells for developing anti-aging drugs, novel ways to facilitate the establishment of stem cell engineering technology, as well as the critical issues of defining and validating the safety and efficiency of anti-aging treatments.

These were just several subjects of the vast and ongoing discourse. There is no doubt that the broad international academic and public consultation for the advancement of the Anti-aging and Disease Prevention field will continue, within the new UNESCO Committee and other frameworks. We hope that the new committee and allied frameworks will be successful in their work promoting healthy longevity for all through education, science and culture.

## References

[1] Jin K, Simpkins JW, Ji X, Leis M, Stambler I (2015). The critical need to promote research of aging and aging-related diseases to improve health and longevity of the elderly population. Aging Dis, 6(1): 1-5. 25657847 10.14336/AD.2014.1210 PMC4306469

[2] Stambler I, Jin K, Lederman S, Barzilai N, Olshansky SJ, Omokaro E, Barratt J, Anisimov VN, Rattan S, Yang S, Forster M, Byles J (2018). Aging health and R&D for healthy longevity must be included into the WHO Work Program. Aging Dis, 9(2): 331-333. 29896422 10.14336/AD.2017.1120 PMC5963354

[3] United Nations, Department of Economic and Social Affairs, Population Division (2019). World Population Prospects: The 2019 Revision. Accessed December 2019. Retrieved from: https://esa.un.org/unpd/wpp.

[4] Stambler I. Life extension: opportunities, challenges, and implications for public health policy. In: Vaiserman A, editor. Anti-aging Drugs: From Basic Research to Clinical Practice. London: Royal Society of Chemistry; 2017, pp. 537-564.

[5] Goldman DP, Cutler DM, Rowe JW, Michaud PC, Sullivan J, Peneva D, Olshansky SJ (2013). Substantial health and economic returns from delayed aging may warrant a new focus for medical research. Health Aff 32(10): 1698-1705. 10.1377/hlthaff.2013.0052 PMC3938188 24101058

